# A novel ternary magnetic nanobiocomposite based on tragacanth-silk fibroin hydrogel for hyperthermia and biological properties

**DOI:** 10.1038/s41598-024-58770-9

**Published:** 2024-04-08

**Authors:** Reza Eivazzadeh-Keihan, Adibeh Mohammadi, Hooman Aghamirza Moghim Aliabadi, Amir Kashtiaray, Milad Salimi Bani, Amir Hossein Karimi, Ali Maleki, Mohammad Mahdavi

**Affiliations:** 1 Catalysts and Organic Synthesis Research Laboratory, Department of Chemistry, Iran University of Science and Technology, Tehran, 16846-13114 Iran; 2 Advanced Chemical Studies Lab, Department of Chemistry, K. N. Toosi University of Technology, Tehran, Iran; 3 Department of Biomedical Engineering, Faculty of Engineering, University of Isfahan, Isfahan, Iran; 4 Mechanical Engineering Faculty, Isfahan University of Technology, Isfahan, Iran; 5 Endocrinology and Metabolism Research Center, Endocrinology and Metabolism Clinical Sciences Institute, Tehran University of Medical Sciences, Tehran, Iran

**Keywords:** Tragacanth hydrogel, Silk fibroin, Biological properties, Hyperthermia, Magnetic nanobiocomposite, Chemistry, Materials science, Nanoscience and technology

## Abstract

This study involves the development of a new nanocomposite material for use in biological applications. The nanocomposite was based on tragacanth hydrogel (TG), which was formed through cross-linking of Ca^2+^ ions with TG polymer chains. The utilization of TG hydrogel and silk fibroin as natural compounds has enhanced the biocompatibility, biodegradability, adhesion, and cell growth properties of the nanobiocomposite. This advancement makes the nanobiocomposite suitable for various biological applications, including drug delivery, wound healing, and tissue engineering. Additionally, Fe_3_O_4_ magnetic nanoparticles were synthesized in situ within the nanocomposite to enhance its hyperthermia efficiency. The presence of hydrophilic groups in all components of the nanobiocomposite allowed for good dispersion in water, which is an important factor in increasing the effectiveness of hyperthermia cancer therapy. Hemolysis and 3-(4,5 dimethylthiazol-2-yl)-2,5-diphenyl tetrazolium bromide assays were conducted to evaluate the safety and efficacy of the nanobiocomposite for in-vivo applications. Results showed that even at high concentrations, the nanobiocomposite had minimal hemolytic effects. Finally, the hyperthermia application of the hybrid scaffold was evaluated, with a maximum SAR value of 41.2 W/g measured in the first interval.

## Introduction

Polymeric biomaterials can be developed to create an artificial matrix that mimics the cell microenvironment, promoting the growth of new tissue. Natural polymers, such as chitosan and hyaluronic acid, are biocompatible and biodegradable materials that can be extracted from biological sources and have found widespread application in the biomedical field due to their ability to mimic the extracellular matrix of native tissues^1–5^.

Tragacanth gum (TG) is a hydrophilic hetero-polysaccharide composed of a main chain consisting of 1,4-linked α-d-galacturonic acid, accompanied by side chains of xylose, arabinose, galactose, and fucose. When TG is combined with PVA (polyvinyl alcohol), has exhibited favorable antibacterial properties in biomaterials. The utilization of tragacanth gum, a natural polysaccharide, in combination with synthetic polymers has resulted in the development of blended hydrogels^6–8^. These hydrogels have demonstrated enhanced wound-healing capabilities when employed as wound coverings. Studies have indicated that the incorporation of TG into wound dressings improves the mechanical properties, elasticity, adhesion, and cell proliferation of the dressings. Notably, TG dressings have been reported to promote burn healing without causing skin toxicity. The hydrolysis of TG into arabinose and glucuronic acid has been observed to induce protein coagulation, thereby facilitating wound healing^9^. Tragacanth gum has been attractive for biomedical applications due to its abundance, biocompatibility, and biodegradability. Nowadays, hydrogels based on polysaccharides have been applied in biomedical applications such as tissue engineering, drug delivery systems, wound dressing, biosensors, and hyperthermia cancer therapy^10^.

Hydrogels are polymeric materials that can absorb water and biological fluids. Natural polysaccharides are great for creating hydrogels for in-vivo applications. Hydrogels are formed by self-assembly of small polymers or macromolecules. Ionic crosslinking agents are less toxic and have been used in hydrogel preparation. TG hydrogel is a polysaccharide hydrogel that is biocompatible, biodegradable, and has high reversibility. However, its poor mechanical properties limit its biomedical applications. Hybridizing TG hydrogel with materials that improve its mechanical properties is effective. Silk fibroin has excellent mechanical properties, biocompatibility, biodegradation, and functionalization, making it useful for innovative applications^11–17^.

This protein is extracted from various sources such as silkworms, spiders, mites, flies, and scorpions^18^. For a long time, silk fibroin was used as a textile fiber. Also, for decades, silk fibroin has been used as a surgical suture material, and is being further expanded for diverse emerging biomedical applications^19–23^. It can be used as a scaffolding material to enhance the adhesiveness and growth ratio of the cells. In addition it is less immunogenic, nontoxic, noncarcinogenic, and has good biocompatibility and mechanical strength^24–28^. According to reports, combining magnetic nanoparticles with polysaccharide hydrogels can improve their biological applications. To design more effective systems based on magnetic nanoparticles in biomedicine applications, all the characteristics of nanoparticles, including surface chemistry, magnetic properties, and toxicity, should be addressed^29^.

Magnetic nanoparticles have been widely used in different therapeutic methods, including targeted drug delivery and hyperthermia. In addition, according to recent applications, these nanoparticles can reduce implant infection and increase tissue growth^29^. Magnetite (Fe_3_O_4_ MNPs) is one of the best magnetic nanoparticles with appropriate properties including antibacterial, and superparamagnetism behavior that showed excellent results in hyperthermia and drug delivery treatment methods^30–33^. Hyperthermia is a low-risk method with minimal side effects on the body which might be a practical alternative for radiotherapy, chemotherapy, and surgery. Therefore, it has attracted the attention of scientists in recent decades^34,35^.

In this research, a new nanocomposite based on TG hydrogel is prepared for biological applications.Tragacanth chains make three-dimensional network by Ca^2+^ ions as a cross-linking agent. Silk fibroin, a natural protein with high biocompatibility, low toxicity, adhesion and cell growth properties, was selected as the second component of the nanocomposite and combined with the hydrogel structure. Finally, magnetic nanoparticles were synthesized in situ in the nanobiocomposite structure. The synthesized nanobiocomposite is designed for biological applications, so natural materials with low toxicity are used. Furthermore, our objective is to create a suitable nanocomposite for hyperthermia therapy. To achieve this, we have utilized a water-swellable hydrogel to enable the homogeneous dispersion of the nanocomposite in a water environment. It really matters that the designed nanocomposite does not settle quickly after dispersion in the aqueous medium because rapid settlement reduces the hyperthermic effect. The water-swelling property of the hydrogel ensures that the nanocomposite settles at a later time, allowing for adequate time for hyperthermia treatment. In addition, the presence of hydrophilic groups such as COOH, –OH and –NH in the structure causes a better dispersion of the nanocomposite in the aqueous media. In order to enhance the efficacy of hydrogels for biological applications, it is imperative to consider their strength, adhesion, and ability to promote cell growth. The purpose of in situ synthesis of magnetic nanoparticles was their good dispersion in the nanocomposite structure because good dispersion of particles is very effective in hyperthermia efficiency. Hemolysis and MTT tests were performed to investigate cytotoxicity. The results of biological tests show it is hemocompatible and has low cytotoxicity. Therefore, it can be an excellent candidate for hyperthermia cancer therapy and in-vivo application.

## Experimental

### Material

All chemicals (solvents and reagents) used in all stages of synthesis including TG (CAS No. 9000-65-1), CaCl_2_ salt (CAS No.10043-52-4), Na_2_CO_3_ (CAS No. 497-19-8), LiBr (CAS No. 7550-35-8), Ethylenediaminetetraacetic acid (EDTA) (CAS No. 6381-92-6), 2-Amino-2-(hydroxymethyl)-1,3-propanediol (Tris base) (CAS No. 77-86-1), FeCl_3_.6H_2_O (CAS No. 10025-77-1), FeCl_2_.4H_2_O (CAS No. 13478-10-9), ammonia (CAS No. 7664-41-7), and dialysis tubing cellulose membrane (14,000 Da) were prepared from Sigma-Aldrich and Merck companies. The silkworm cocoons were purchased from native breeders. Also, the Mili-Q ultra-pure water was applied.

### Preparation method

#### Preparation of TG hydrogel

According to previous works^36^, 1.0 g of TG gum was dissolved in 50.0 mL of distilled water and stirred for 3 h at 60 °C to obtain a homogeneous solution. After that, 3.90 g of NaOH was added to the TG solution and stirred for 1 h to convert carboxylic acid groups to carboxylate ions. Then, the previously prepared solution was added drop by drop to 10.0 mL (0.20 M) solution of CaCl_2_ (as a cross-linking agent). Afterward, the mixture solution was stirred for 1 h at room temperature. Then, the prepared hydrogel was neutralized to pH = 7 by the solution of HCl (0.10 M). In this way, the hcl solution is added drop by drop and the pH of the solution is checked regularly until it reaches pH = 7. Finally, it was washed several times with distilled water to remove the excess amount of HCl. The prepared hydrogel was kept at − 70 °C for 24 h and put into the freeze-dryer device.

#### SF extraction

Accordingly, in previous research work^19,20^, three silkworm cocoons were cut into tiny pieces. Then, the sodium carbonate solution (0.114 M) was prepared in distilled water and tiny pieces of silkworm cocoons were added to it. After that, the mixture solution was boiled for 2 h. Next, the silk fibers were washed several times with distilled water and dried at room temperature overnight. To prepare the dialysis membrane was boiled in 200.0 mL of distilled water containing 0.242 g of tris and 0.058 g of EDTA for 2 h. After that, 0.52 g of dried silk fibers were dissolved in the LiBr solution (9.30 M) and stirred for 2 h at 60 °C. Finally, the prepared solution was added to the dialysis membrane and put in a 1.0 L of distilled water for 2 days at room temperature.

#### Preparation of TG hydrogel/SF/Fe_3_O_4_

To enhance the mechanical strength, adhesiveness, and magnetic properties of TG hydrogel without any more toxicity, the modifying of TG hydrogel with SF and Fe_3_O_4_ MNPs was performed in situ synthesis as follows:

Firstly, in a round-bottom flask, 10.0 mL of extracted SF and 10.0 mL of TG hydrogel solution were mixed together under continuous stirring for 10 min at room temperature. Then, 0.74 g of FeCl_2_·4H_2_O and 1.64 g of FeCl_3_·6H_2_O were dissolved in 50.0 mL of distilled water and added to the previous mixture solution under an N_2_ atmosphere. Ammonia was added dropwise until a pH of approximately 12 was achieved. The stirring was continued for 2 h at 70 °C. Finally, the TG hydrogel/SF/Fe_3_O_4_ nanobiocomposite was magnetically collected, washed several times with deionized water and ethanol, and dried at 60 °C.

All analysis including Fourier-transform infrared spectroscopy (FTIR), X-ray diffraction (XRD), energy-dispersive X-ray (EDX), field-emission scanning microscopy (FESEM), thermogravimetric analysis (TGA), vibrating-sample magnetometer (VSM), and Transmission electron microscopy (TEM) was taken to evaluate the structure of the prepared magnetic nanobiocomposite.

### Fourier-transform infrared spectroscopy (FT-IR)

FT-IR is an analysis to identify chemical bonds in molecules by producing an infrared absorption spectrum. All of the bonds and functional group in the structure of magnetic nanobiocomposite was evaluated by Fourier-transform infrared spectrometer (Perkin Elmer Spectrum RX-1).

### X-ray diffraction (XRD)

X-ray diffraction (XRD) is a widely used technique to evaluate the crystallinity and structure of solid samples. It is used for phase identification of a crystalline material. The XRD pattern of this sample was taken by the Brucker X-ray diffractometer device (D8 Advanced Model, Germany). This device contains a Lynxeye detector (0D mode) with Cu-Kα radiation (λ = 0.154 nm, 40 kV, 40 mA)^21^.

### Field-emission scanning microscopy (FE-SEM)

The FE-SEM analysis is an analytical technique used to investigate molecular surfaces and the morphology of samples. As mentioned above, The FE-SEM analysis was taken by electron scanning microscope (TESCAN—Mira III model).

### Energy-dispersive X-ray (EDX)

The EDX analysis is an analytical technique used for the elemental analysis of the sample. The EDX analysis was performed by an EDX detector (ESCAN MIRA II SAMX detector model) which attached to the electron scanning microscope (TESCAN—Mira III model).

### Thermogravimetric analysis (TGA)

TGA is an analysis used to determine thermal stability and fraction of volatile components of the sample by controlling the weight change that occurred with increasing temperature. This analysis was done by using the Bahr-STA 504 instrument (Germany) with 5 mg of sample in alumina pans under an argon atmosphere with a 1 L/h flow rate and a constant heating rate (10 °C/min)^24^.

### vibrating-sample magnetometer (VSM)

The VSM analysis is a scientific instrument that measures magnetic properties based on Faraday’s Law of Induction. This analysis was performed by LBKFB model-magnetic Kashan Kavir (5000 Oe) instrument^20^.

### Hemolytic assay

A hemolysis assay was performed based on the method presented in our previous studies. Primarily, after completing the informed consent form, fresh blood samples were taken from a volunteer with blood type O. Then, human red blood cells (RBCs) were washed and diluted with physiological serum in a ratio of 1:50. Next, 100 µL of it was poured onto a 96-well V-shaped bottom plate (Citotest, China). Then, each well received 100 µL of dispersed nanobiocomposite in physiological serum with concentrations of 0.25, 0.5, 0.75, 1 and 2 mg/mL. Triton X-100 and physiological serum were applied as positive and negative controls, respectively. After 2 h of incubation at 37 °C, the plate was centrifuged at 2000 rpm for 10 min. Finally, the supernatant of each well was transferred to the flat bottom plate and the optical density (OD) was measured at 405 nm by the ELISA reader (Biohit, Finland)^23,37^. The hemolysis percentage was calculated by the following formula^19^:

$$ Hemolysis\% = \left[ {\frac{mean\;OD\;of\;sample - mean\;OD\;of\;negative\;control}{{mean\;OD\;of\;positive\;control - mean\;OD\;of\;negative\;control}}} \right] \times 100 $$

Ethical issues:

This study was performed in accordance with the principles outlined in the Declaration of Helsinki. Also, the experimental methods and the procedure for obtaining informed satisfaction were approved by the Ethics Research Committee of the Pasteur Institute of Iran.

#### MTT assay

The toxicity of the TG hydrogel/SF/Fe_3_O_4_ nanobiocomposite against the BT549 (breast cancer) cell line and HEK293T (human embryonic kidney) cell line was determined using MTT assay, according to our previous studies. First, both cell lines were provided by the Pasteur Institute of Iran. Next, the proper culture medium contains Dulbecco’s Modified Essential Medium and Ham’s F-12 Medium (DMEM/F12), 10% fetal bovine serum (FBS), and 1% Penicillin–Streptomycin (Pen-Strep) was prepared and the cells were cultured at 5 × 10^3^ cells/well in 96 well plates. Then, serially dilutions of nanobiocomposite with concentrations of 0.015, 0.031, 0.062, 0.125, 0.25, 0.5, 0.75, 1, 1.25, 1.5 and 1.75 mg/mL were added to each well and incubated for 48 h and 72 h. Cisplatin (Sigma-Aldrich, MO, United States) and the culture medium without any additive were used as the positive and negative controls, respectively. The cells were then treated with MTT solution (Sigma, USA) and incubated for 4 h at 37 °C. Next, 1% SDS was added to the wells and incubated for 16 h at 37 °C. Finally, the optical density of samples was measured at 550 nm using a microplate reader spectrophotometer (BioTeK, USA). All tests were done in triplicate^20,38^. The percentage of toxicity and cell viability were calculated as follows^21^:

1 $$ Toxicity\% = \left( {1 - \frac{mean\;OD\;of\;sample}{{mean\;OD\;of\;negative\;control}}} \right) \times 100 $$

2 $$ Viability\% = 100 - Toxicity\% $$

#### Statistical analysis

Statistical analysis for the comparison all biocompatibility and hemocompatibility results was accomplished by a t-test by SPSS Statistics 22.0 software (SPSS Inc. Chicago, IL, USA). The values of *P* ≥ 0.05 (*), *P* ≤ 0.05 (**) and *P* ≤ 0.001 (***) were considered statistically insignificant, significant and very significant, respectively.

#### Swelling properties

Freeze-dried nanobiocomposite was immersed in ultra-pure water (UPW) at 25 °C for 48 h. Surplus UPW was then removed from the surface of sample and the wet weight of the nanobiocomposite was determined. The swelling ratio and the water uptake in the sample were calculated as follows:

3 $$ Swelling\;ratio \left( \frac{g}{g} \right) = \left[ {\frac{{W_{s} - W_{d} }}{{W_{d} }}} \right] $$

4 $$ Water\;uptake \left( \% \right) = \left[ {\frac{{W_{s} - W_{d} }}{{W_{d} }}} \right] \times 100 $$

In this formula, W_s_ and W_d_ are the weights of dried and swollen nanobiocomposite, respectively^39^.

#### Biodegradability assay

For degradation experiments, TG hydrogel/SF/Fe_3_O_4_ magnetic nanobiocomposite was placed into PBS at pH = 7.4 and 37 °C. The buffer solution was refreshed every 3 days. This test was performed up to 10 days and at the selected time points, three samples of nanobiocomposite were removed from the buffer and weighed wet after surface wiping. Afterward, they were rinsed with UPW and dried in a vacuum oven at 37 °C for 24 h. Water absorption and weight loss were calculated according to these formulas:

$$ Water\;absorption \left( \% \right) = \left[ {\frac{{W_{a} - W_{0} }}{{W_{0} }}} \right] $$

$$ Weight\;loss \left( \% \right) = \left[ {\frac{{W_{0} - W_{t} }}{{W_{0} }}} \right] $$

where W0 is the starting dry weight, Wa is the wet sample weight after removal from the solution, and Wt is the dry sample weight after removal^40^.

## Result and discussion

### Preparation of TG hydrogel/SF/Fe_3_O_4_ bionanocomposite

**Scheme 1:**
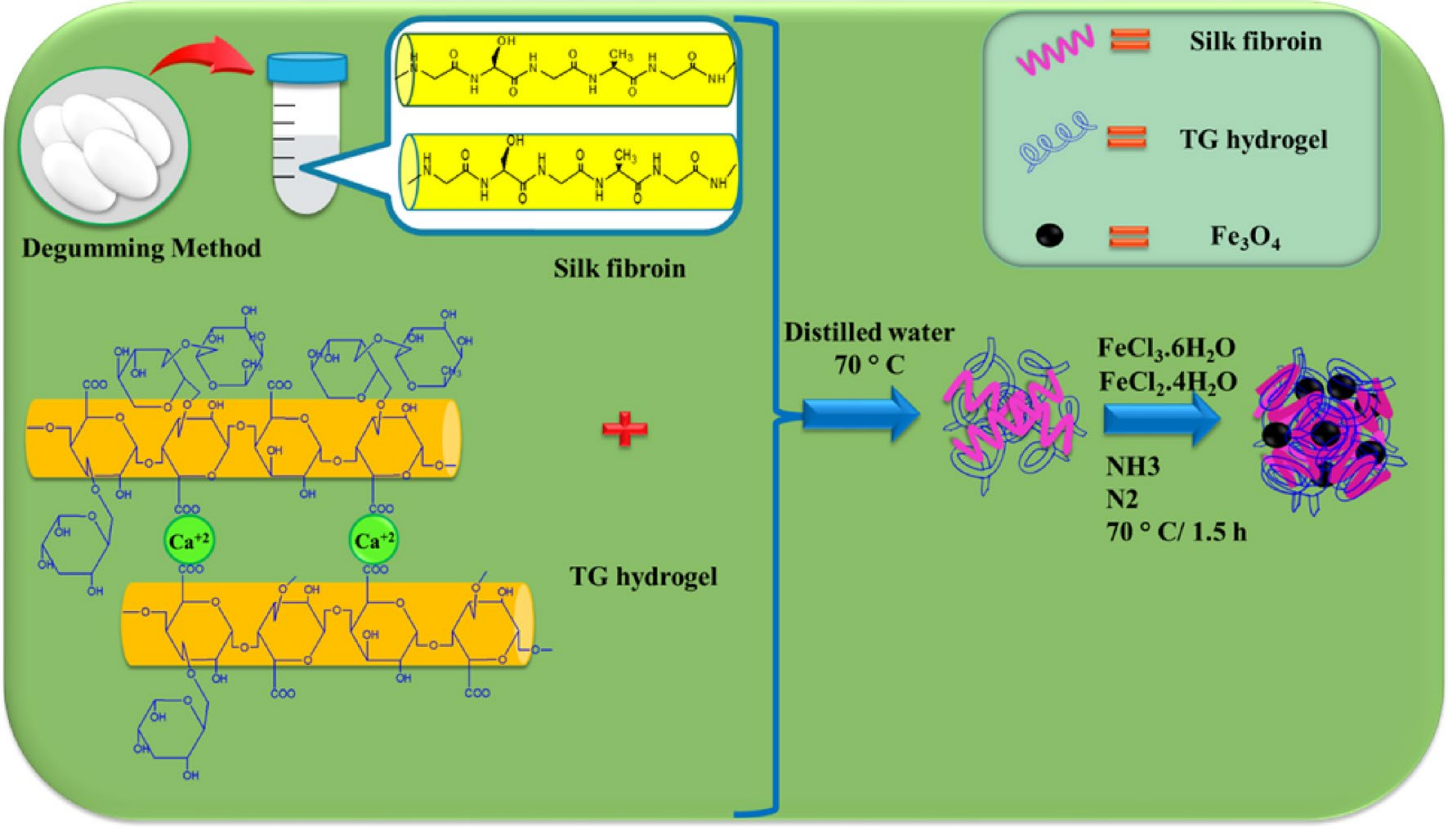
Synthesis preparation of TG hydrogel/SF/Fe_3_O_4_ magnetic nanobiocomposite scaffold.

In this research work, a novel multi-functional magnetic nanobiocomposite based on natural polymers with low toxicity such as polysaccharide (TG), natural protein (SF), and Fe_3_O_4_ MNPs. All synthesis steps are shown in Scheme 1. This magnetic nanobiocomposite was designed for several purposes including low toxicity, hemocompatibility, and high magnetic properties that can be applied effectively for hyperthermia cancer therapy. At first, tracagans strands are connected to each other through bonding interactions between Ca^2+^ ions and –COO^−^ group and form the hydrogel network. In fact, calcium ions act as a cross-linking agent. After that, natural silk fibroin protein was combined with tragacanth hydrogel through establishing hydrogen bonds to increase mechanical strength, adhesion and cell growth. In the final step, magnetic iron nanoparticles were added in-situ to the prepared nanocomposite. The primary motivation behind conducting in-situ synthesis of magnetic nanoparticles was to achieve effective dispersion within the nanocomposite structure. This dispersion plays a crucial role in enhancing the efficiency of hyperthermia therapy.

### FT-IR spectrum and TGA analysis

**Figure 1:**
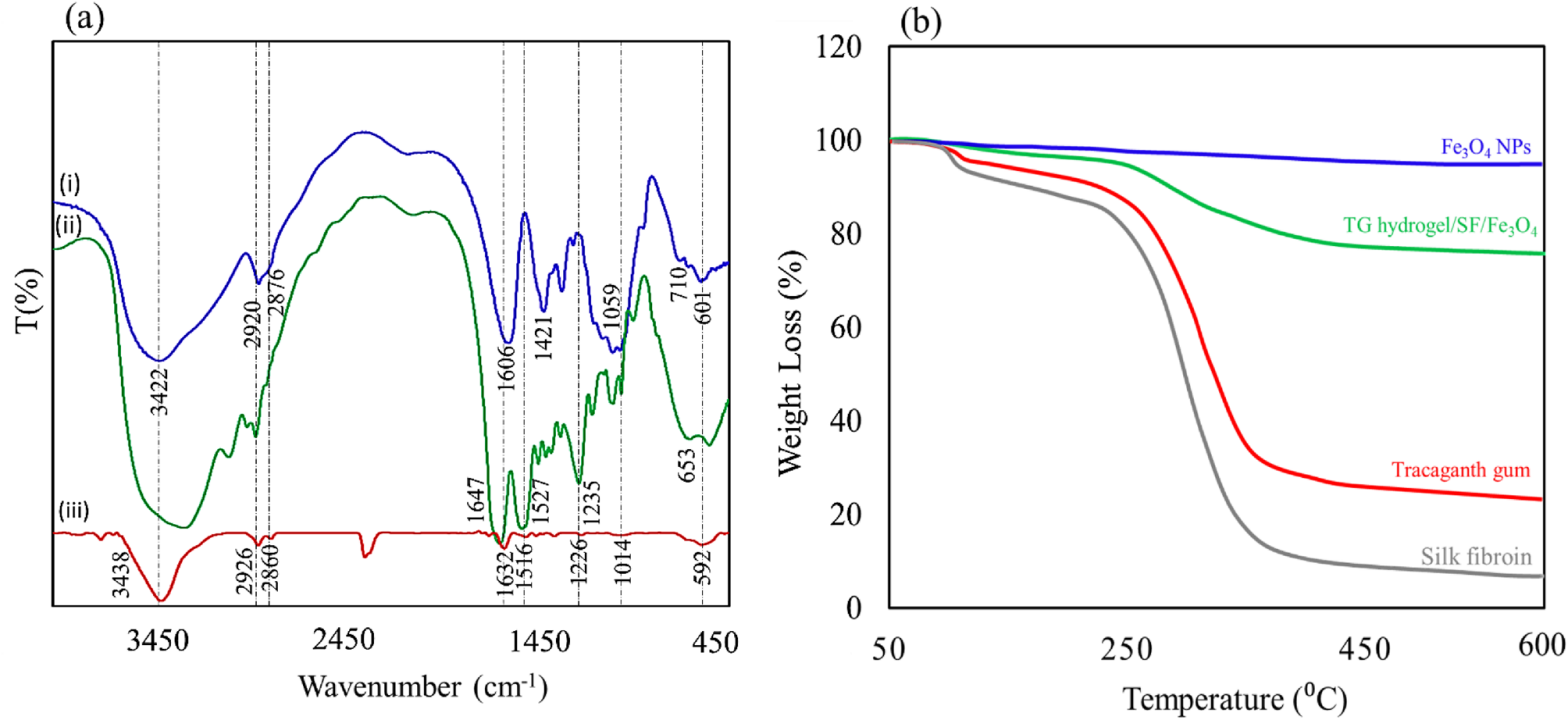
FT-IR spectrum (**a**) of TG hydrogel(i), SF(ii), and TG hydrogel/SF/Fe_3_O_4_ and TGA analysis (**b**) of TG hydrogel (red curve), SF (gray curve), Fe_3_O_4_ MNPs (blue curve), and TG hydrogel/SF/Fe_3_O_4_ (green curve) nanobiocomposite.

The FT-IR spectrum was evaluated in Fig. 1a to characterize the functional groups of the TG hydrogel(i), SF(ii), and prepared nanobiocomposite(iii). The FTIR spectrum(i) revealed several distinctive peaks, including a peak at 3422 cm^−1^ which indicated the presence of -OH functional group of carbohydrates. The peaks at 2920 and 2876 cm^−1^ were indicative of the aliphatic –CH vibration. Furthermore, the peaks at 1606 and 1421 cm^−1^ represented galacturonic acid and the –C=O bending vibration, respectively. Additionally, the peaks at 1059, 710, and 601 cm^−1^ were indicative of the –C–O–C– aliphatic stretching vibration, glycosidic linkage, and pyranose of TG. These findings suggest that tragacanth gum possesses a complex molecular structure with various functional groups, which makes it a promising material for advanced biomedical applications^41^. The FTIR spectrum(ii) shows the functional groups of the SF structure. The SF spectrum (Fig. 1a(ii)) indicated the presence of amide I (C=O stretching band) at a vibration band of 1647 cm^−1^. Additionally, the vibration bands at 1527 and 1235 cm^−1^ were attributed to amide II (secondary N–H bending) and amide III (C–N and N–H functionalities), respectively^42,43^. The FTIR spectrum (iii) shows the FTIR spectrum of TG hydrogel/SF/Fe_3_O_4_ nanobiocomposite. A broad absorbance band appeared around 3438 cm^−1^, which can be attributed to the stretching vibration of O–H groups in TG. The asymmetric and symmetric stretching vibrations of aliphatic groups (methyl and methylene) appeared at 2926 cm^−1^ and 2860 cm^−1^, respectively. The assigned absorption bonds at 1632 cm^−1^ and 1460 cm^−1^ were attributed to the asymmetrical and symmetrical stretching vibration of the –COO^−^ group which has a good match with the spectrum related to Trakagans hydrogel. Also, an absorption bond at 1014 cm^−1^ represented the stretching vibration of the C–O bond in the structure of TG^44^. Also, the three absorption peaks of the SF structure are well defined at 1632, 1516, and 1226 cm^−1^ which are related to the stretching vibration mode of amide I, N–H bending vibration mode of amide II, and the C–N stretching vibration of the mode of amide III^22,23^. Finally, an absorption peak around 592 cm^−1^ was related to the stretching vibration of the Fe–O bond which proved the presence of Fe_3_O_4_ MNPs in the structure of the prepared magnetic nanobiocomposite^45^. The thermal stability of TG (red curve), SF(gray curve), Fe_3_O_4_ MNPs(blue curve), and TG hydrogel/SF/Fe_3_O_4_ (green curve) were investigated by TGA analysis that was shown in Fig. 1b. The TGA curves of Fe_3_O_4_ microspheres show that weight loss over temperatures ranging from 50 to 600 °C is about ~ 3%, which is attributed to the escape of physically adsorbed water or/and structure water on the surface. For TG , the weight loss at temperatures below 200 °C is about ~ 5%, which is related to the release of moisture on the surface of the TG structure. For the TGA curve of TG, in the second step, at the temperature of about 240–600 °C, degradation occurs about ~ 73%. This decomposition may be due to the highly branched heterogeneous structure of GT that possesses the dissipation of side chain groups, such as acidic or ester groups^46,47^. The thermogravimetric curve of SF hydrogels are shown in Fig. 1b (gray curve). The initial weight loss (~ 15%) of SF hydrogels at around 90–100 °C is due to loss of water. The second weight loss (~ 75%) took place within the temperature range between 250 and 410 °C and is associated with the breakdown of side chain groups of amino acid residues as well as the cleavage of peptide bonds. In Fig. 1b (green curve), the sample test showed a characteristic of two-step thermal degradation. The first step (5% loss weight) happened at about 91 °C which attributed to the degradation of molecular water inside the hydrogel matrix. The second stage accrued at around 228–427 °C thermal range due to degradation of the TG hydrogel and SF’s side-chain groups^48^. After that, the mass of this nanobiocomposite remained constant with an increase in temperature.

### XRD and VSM analysis

**Figure 2:**
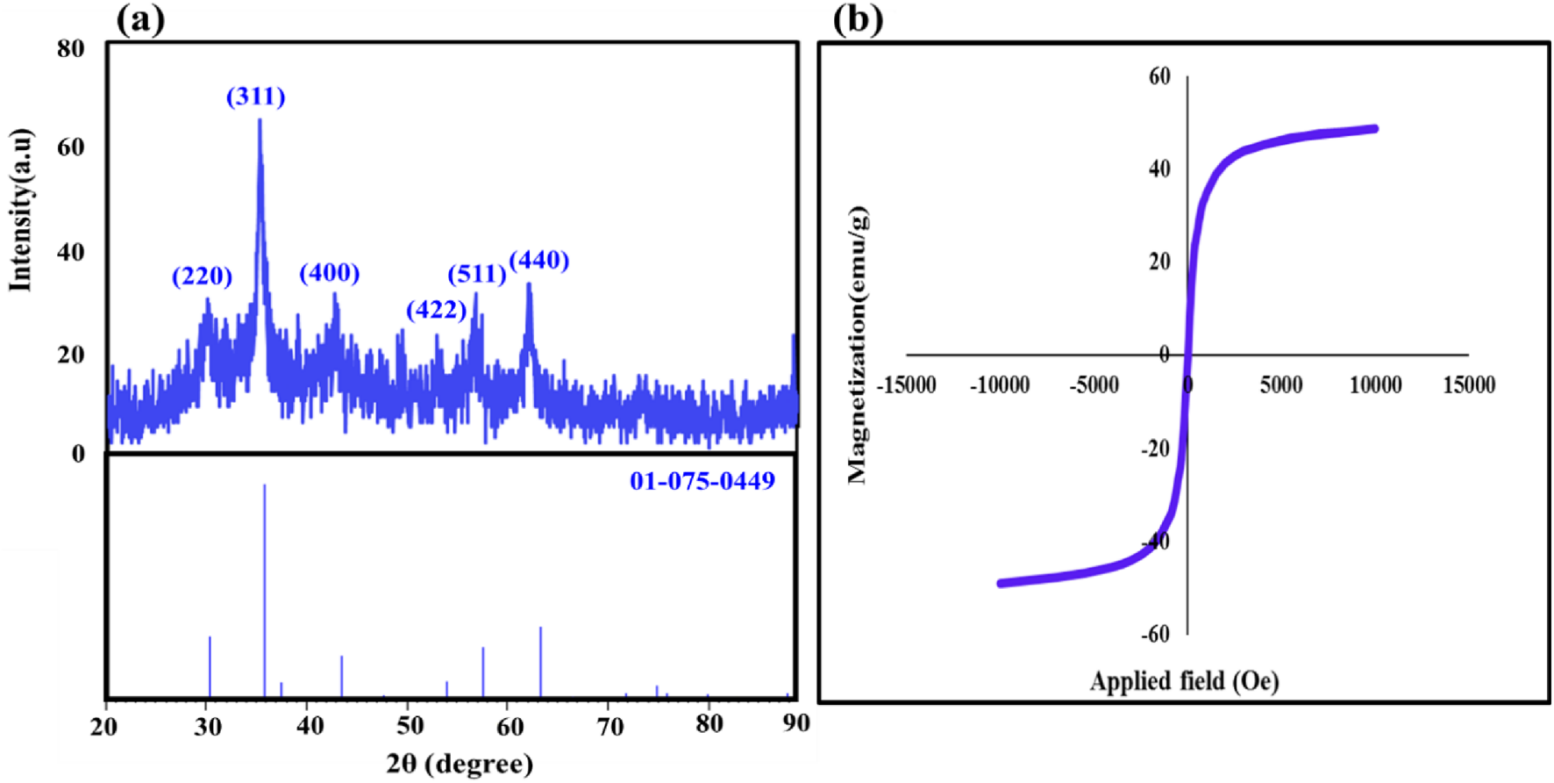
The XRD pattern (**a**) and room-temperature M–H curves of the TG hydrogel/SF/Fe_3_O_4_ (**b**).

The crystal structure of the magnetic nanoparticles was identified by X-ray diffraction pattern, shown in Fig. 2a. The principal characteristic peaks for Fe_3_O_4_ MNPs are demonstrated at 2θ = 30.30°, 35.47°, 43.08°, 53.44°, 57.31°, and 62.93° which attributed to the Miller indices (220), (311), (400), (422), (511), and (440), plane in the crystalline structure, respectively (JCPDS card No. 01-075-0449) . This conformation indicated that the Fe_3_O_4_ MNPs magnetic nanoparticles were pure. In addition, a broadening is observed at the end of the Fe_3_O_4_ peaks, which is related to the amorphous parts of the nanocomposite, including TG hydrogel and SF. The magnetic property of the produced TG hydrogel/SF/Fe_3_O_4_ magnetic nanocomposite was investigated using vibrating-sample magnetometer (VSM) analysis, shown in Fig. 2b. Typically, discernible fluctuations in saturation magnetization (Ms) between functionalized and unfunctionalized magnetic nanoparticles can be ascertained through the use of a vibrating-sample magnetometer. The magnetic characteristics of these particles can be influenced by various factors, including the iron-group crystalline structure, shell thickness, core size, and interparticle and intraparticle interactions^49^. Reportedly, the saturation magnetization of Fe_3_O_4_ nanoparticles was 76.20 emu/g^50^. This value was obtained for the final nanocomposite 48.76 emu/g. The decrease in the value of saturation magnetization in relation to nanoparticles only indicates the successful dispersion of these nanoparticles in the polymer matrix. Previous research has shown that covering nanoparticles with polymer structures can have a significant effect on their saturation magnetization in the applied magnetic field. As previously noted, the thickness of the shell surrounding Fe_3_O_4_ nanoparticles plays a crucial role in reducing their magnetism. This study involved the use of a hydrogel network and protein strands to cover the Fe_3_O_4_ magnetic nanoparticles, resulting in a significant decrease in their saturation magnetism. However, after the dispersion of these nanoparticles in the structure of the nanocomposite, the saturation magnetization value is still acceptable, which is required for the high efficiency of the nanocomposite in hyperthermia therapy.

### EDX analysis

**Figure 3:**
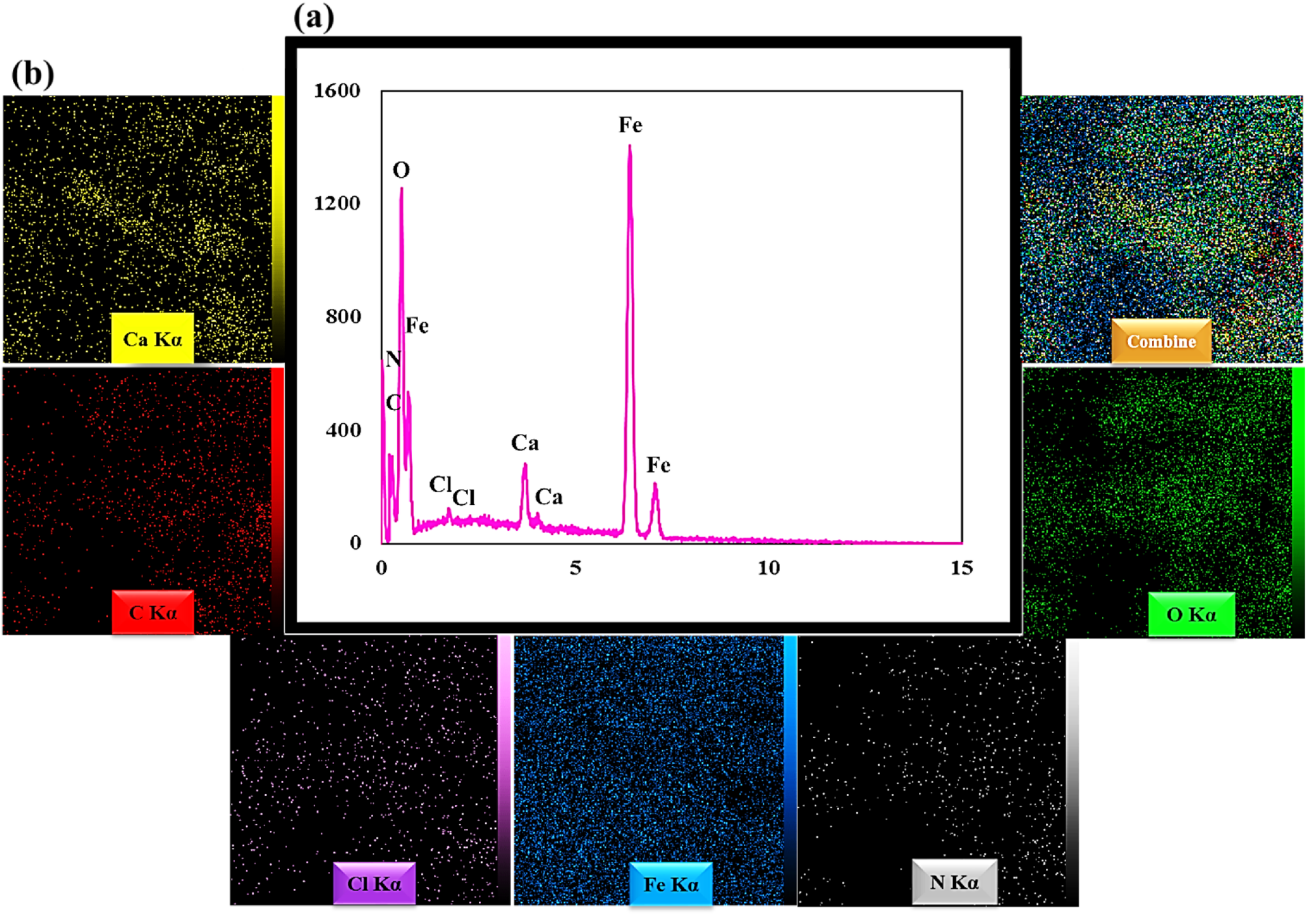
EDX spectrum (**a**), elemental mapping images of TG hydrogel/SF/Fe_3_O_4_ nanobiocomposite scaffold (**b**).

The qualitative detection technique EDX was used to identify the structural elements of the prepared nanobiocomposit. These results are well shown in Fig. 3a. Initially, the tracaganes hydrogel was synthesized by utilizing tracaganes polymer strands and CaCl_2_ cross-linking agent. The polymer strands formed a robust three-dimensional hydrogel network through electrostatic interactions with Ca^2+^ ions. The successful preparation of the hydrogel and its incorporation within the nanocomposite structure was confirmed by the detection of carbon, oxygen, and calcium peaks, serving as compelling evidence of the efficacy of the process. Following the initial synthesis of the tracaganes hydrogel, the next stage involved the integration of silk fibroin protein strands to enhance mechanical strength and promote cell adhesion. The observation of carbon, oxygen, and nitrogen peaks within the resulting nanocomposite structure can be attributed to the presence of silk fibroin and its associated molecular structure. This development represents a significant advancement in the field of biomaterials research. In the last step, Fe_3_O_4_ MNPs were dispersed in the gel matrix. The iron peaks identified in the spectrum proved the presence of Fe_3_O_4_ MNPs. It should be noted that the insignificant peak of chlorine ions can be due to the small amount of chlorine trapped in the structure. In Fig. 3b, the homogeneous distribution of particles is well shown by elemental mapping images.

### FE-SEM and TEM images

**Figure 4:**
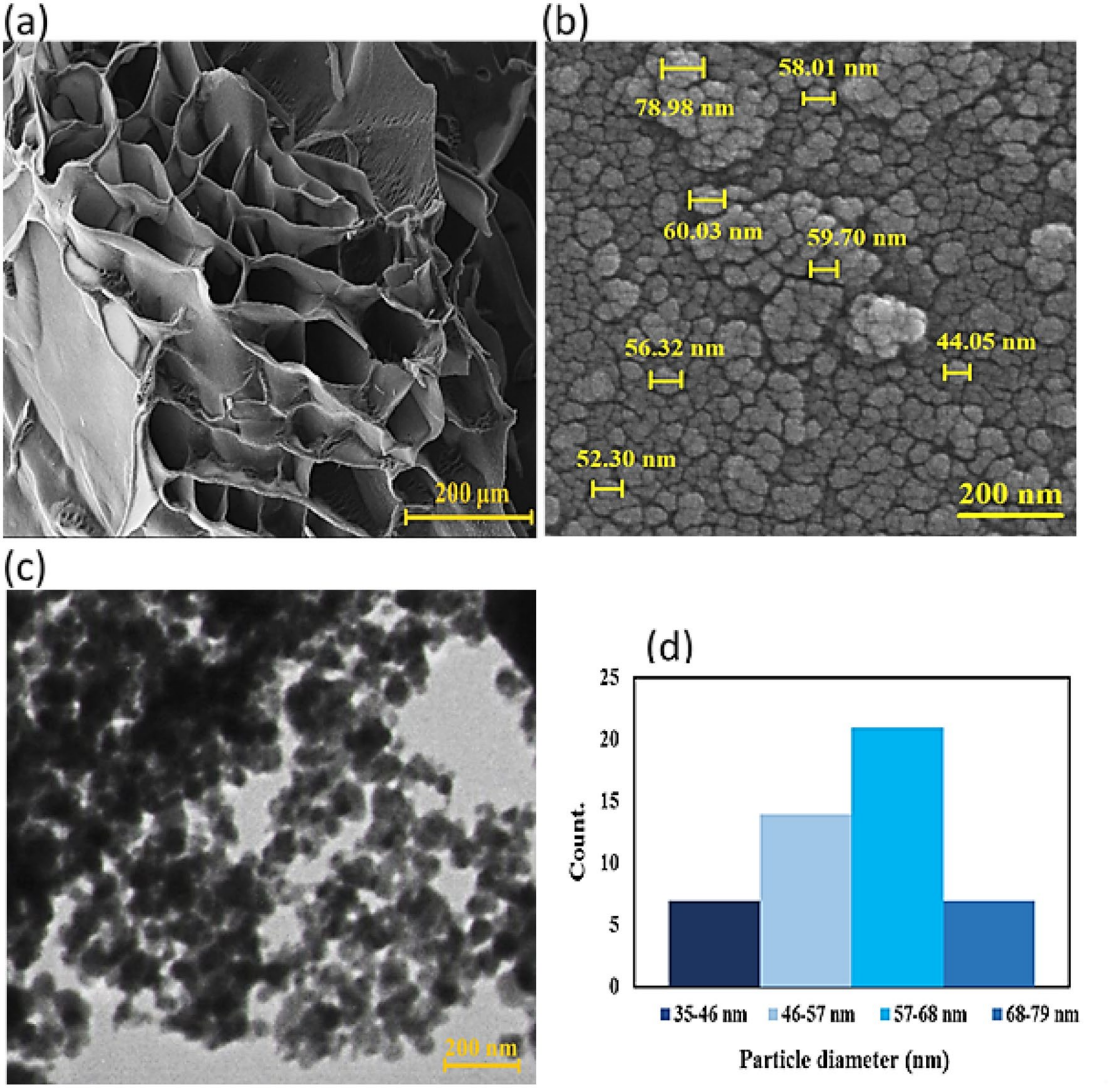
The FE-SEM image of TG hydrogel (**a**), TG hydrogel/SF/Fe_3_O_4_ magnetic nanobiocomposite (**b**), TEM image of TG hydrogel/SF/Fe_3_O_4_ magnetic nanobiocomposite(**c**), and the particle size histogram of TG hydrogel/SF/Fe_3_O_4_ nanobiocomposite scaffold (**d**).

The FE-SEM image was used to evaluate the morphology, size, and structure of the TG hydrogel/SF/Fe_3_O_4_ nanobiocomposite scaffold, shown in Fig. 4a, b. The porous structure of the hydrogel is clearly visible in Fig. 4a. This figure revealed a macroporosity structure of the freeze-dried TG hydrogel with pore size around 100 ± 24 µm. Upon modification with SF polymer strands and synthesis of Fe_3_O_4_ nanoparticles within the matrix, the hydrogel cavities become fully covered by these two components, resulting in a rough and uneven surface. These images confirm the successful synthesis of the designed nanocomposite. In fact, Fe_3_O_4_ MNPs have a spherical morphology and are well-loaded on the TG hydrogel/SF surface and inside it (Fig. 4b). The Fe_3_O_4_ nanoparticles were synthesized in situ, resulting in their optimal dispersion within the hydrogel matrix, a fact that is evident in its transparent form. Additionally, Fig. 4c depicts the dispersion of magnetic nanoparticles throughout the hydrogel structure in the final nanocomposite as observed by the TEM image. In addition, The particle size histogram of TG hydrogel/SF/Fe_3_O_4_ nanobiocomposite showed that the particle size histogram showed that most of the particles had a size between 57 and 68 nm (Fig. 4d).

### Hemocompatibility

**Figure 5:**
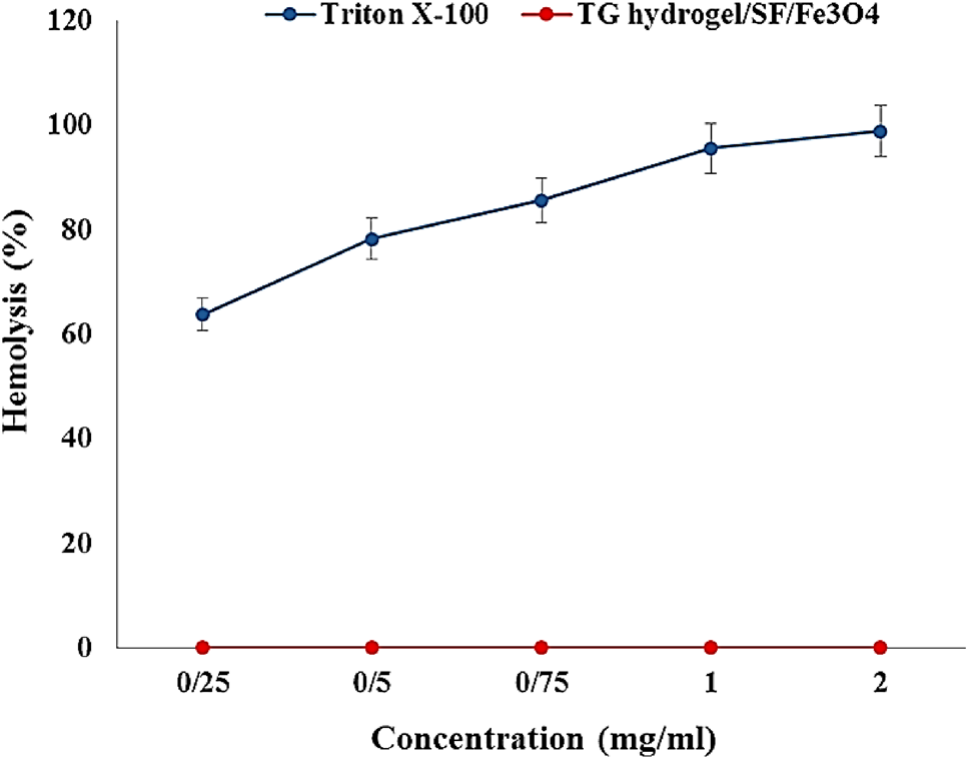
Hemolysis percentage graph of TG hydrogel/SF/Fe_3_O_4_ and Triton X-100 (positive control) with different concentrations (very significant compared to the positive control group, $P\le 0.001$).

Hemolytic activity is an important parameter for materials that come into direct contact with blood^51^. According to the ISO standard (document 10 993-5 1992), when a hemolysis index of a substance is less than 5%, it is considered safe^52^. Results illustrated that TG hydrogel/SF/Fe_3_O_4_ nanobiocomposite has almost no hemolytic effect even at the highest concentration. Instead, Triton X-100 which was used as a positive control, was hemolyzed 100% of RBCs at the same concentration (Fig. 5). The Results are the average of three independent experiments. Based on the results, it can be said that this nanobiocomposite is fully hemocompatible.

### Cytotoxicity

**Figure 6:**
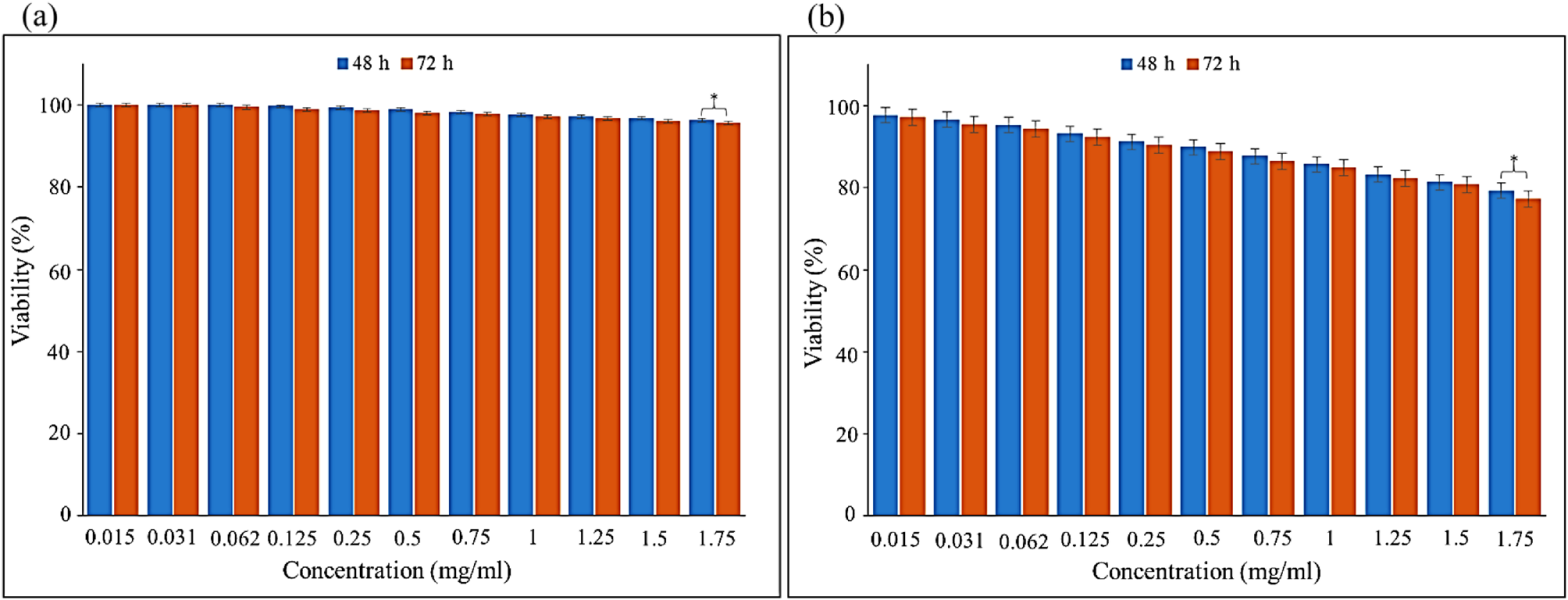
This illustration shows the viability percentage of HEK293T cells (**a**) BT549 cells (**b**) after treatment with TG hydrogel/SF/Fe_3_O_4_ nanobiocomposite at days 2 and 3(* = insignificant, *P* ≥ 0.05).

The results showed that, the viability percentage of HEK293T cells treated with a concentration of 1.75 mg/mL of TG hydrogel/SF/Fe_3_O_4_, after 2 and 3 days was 96.24 ± 0.45% and 95.53 ± 0.64%, respectively (insignificant compared to the negative control, *P* ≥ 0.05), whereas for the BT549 cells, this value was 79.22 ± 1.02% and 77.25 ± 1.23% respectively (Fig. 6a and b) (significant compared to the negative control, *P* ≤ 0.05). So, this synthesized nanobiocomposite is biocompatible with the HEK293T cell line. Also, it has been able to inhibit the growth of BT549 cells and reduce their survival rate.

### Evaluation of swelling properties and biodegradability of nanocomposite

**Figure 7:**
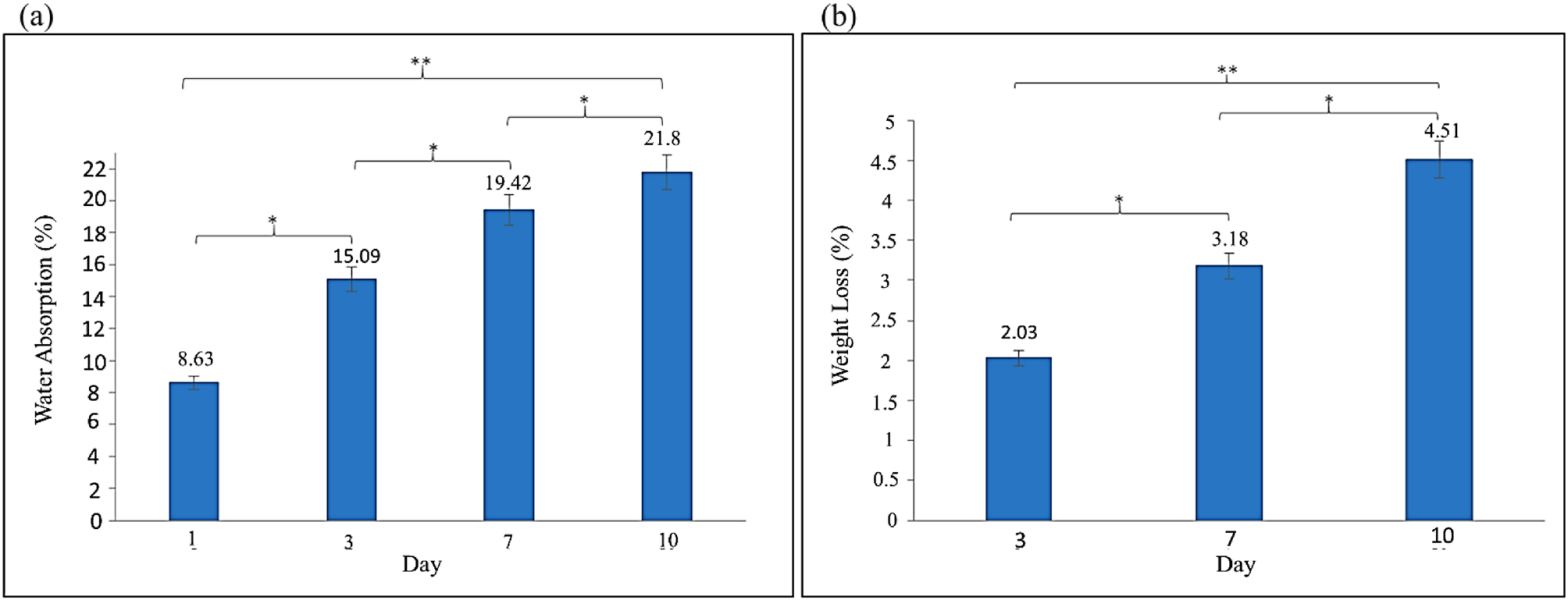
The water absorption (**a**) and The weight loss (**b**) of TG hydrogel/SF/Fe_3_O_4_ nanobiocomposite soaked in PBS for various periods (* = insignificant, *P* ≥ 0.05—** = significant, *P* $\le$ 0.05).

The water-binding ability was measured using the swelling ratio and water uptake. The swelling ratio and water uptake of the TG hydrogel/SF/Fe_3_O_4_ magnetic nanobiocomposite were about 9.4 and 82.12%, respectively. Also, Fig. 7a and b show the water absorption and weight loss of TG hydrogel/SF/Fe_3_O_4_ nanobiocomposite soaked in PBS for various periods. Water absorption of the nanobiocomposite increased throughout the entire incubation period. Also, weight loss of nanobiocomposite occurred very slowly, without appreciable weight change throughout the degradation period.

### Hyperthermia application

There are two methods for measuring heating efficiency, namely thermal dose and specific absorption rate (SAR). In order to reduce the amount of heat required for comfort, it is necessary to produce MNPs with a high SAR. When MNPs are exposed to alternating magnetic fields, they can generate heat through four different processes, including hysteresis, eddy current, Néel or Brownian relaxation, and frictional losses. However, Néel or Brownian relaxation is the most significant in superparamagnetic MNPs. This occurs when the magnetic moment oscillates, causing the domain walls to shift and heat to be produced. The magnetic moments then relax when the magnetic field is removed, either through Néel relaxation or Brownian relaxation, which occurs when each particle spins around its axis.

The contribution of each mechanism depends on various factors, in particular, the frequency of the magnetic field and the particle size. In particular, the effect of hysteresis loss would be more significant in multi-domain magnetic particles of larger size, while for smaller single-domain magnetite nanoparticles where superparamagnetic behavior can be observed, Neel and Brownian relaxation mechanisms are dominant. In such particles, heat loss is caused by the rotation of magnetic moment. If the particle is rotated as well, the shear stress in the fluid is responsible for heat generation (Brownian relaxation loss). Otherwise, while the particle is fixed, heat is dissipated by the inversion of the magnetic moment (Neel relaxation loss)^53–56^.

MNPs’ specific absorption ratio (SAR), which is the rate of heat production, is used to measure how well MNPs heat. SAR is a globally defined parameter to evaluate the effectiveness of MNPs in generating heat during exposure to an alternating magnetic field. The core variable in SAR is the rate of the temperature change created by a nanoparticle and is equal to the temperature difference divided by the time period in which the measurement is performed (∆T/∆t). To have a more meaningful interpretation, it is multiplied by the constant term (C/m) which is then defined as the power of the nanoparticle to increase the temperature.

**Figure 8:**
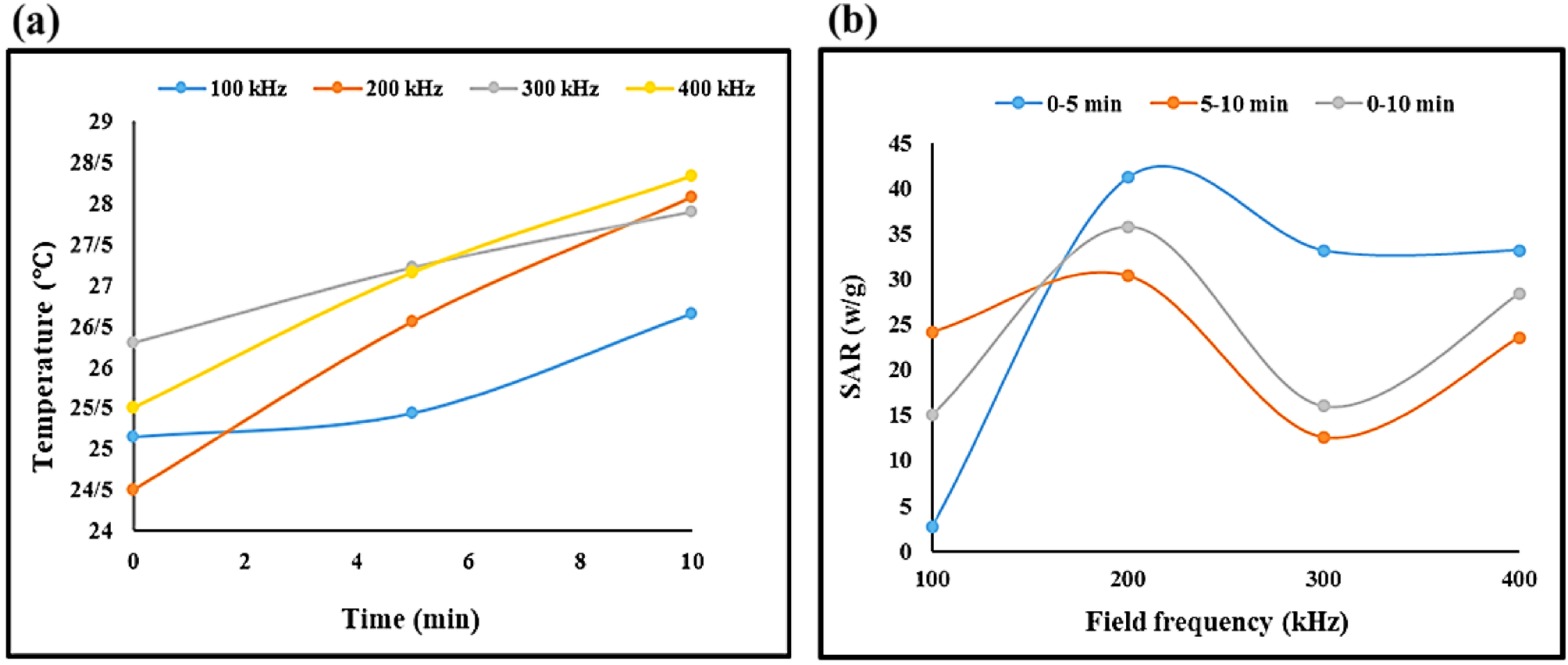
Heating profile (**a**) and SAR as a function of field frequency (**b**) of TG hydrogel/SF/Fe_3_O_4_ magnetic nanobiocomposite.

Initially, synthetic MNPs are formed using chemical methods. A calibrated weight scale and heater with great precision are prepared beforehand. The material used as the core and the one for the coating is mixed using a stirrer. Meanwhile, nitrogen gas is used to prevent any unwanted oxidation. Then an increase in temperature and PH occurs, in order to speed up the desired reaction and the mixture is stirred for several hours under this condition. Finally, the MNPs are collected by an external magnet and washed with deionized water to remove the unreacted materials. During the experiment, the temperature change is calculated in a predetermined time interval under various field frequencies and concentrations of MNPs by a hyperthermia device. A thermocouple is utilized to measure the temperature of the fluid surrounding the MNPs. Equation 5 is used to calculate the SAR value^57^.

5 $$ SAR = \frac{C}{m}\frac{\Delta T}{{\Delta t}} $$

where C is the fluid’s specific heat capacity, m is the concentration of MNPs, and ∆T is the temperature change throughout the time interval ∆t. In this research, MNP heating profiles were determined. A magnetic field oscillated around 1 mg/mL of samples at 24 °C. At 100 kHz, 200 kHz, 300 kHz, and 400 kHz field frequencies with constant field intensity were studied. During the 10-min exposure, the surrounding fluid temperature was recorded every five minutes. As seen in Fig. 8a, the magnetic field caused a fast rise in temperature. In the first time period, 300 kHz saw a 2.3 °C temperature rise. The values for 100 kHz, 200 kHz and 400 kHz are 1.5 °C, 0.5 °C and 1.5 °C, respectively. As a result, there is an increase in the temperature as the field frequency rises in the first five minutes. On the other hand, during the second time period, the field frequency of 300 kHz was responsible for the greatest increase in temperature, which was 4.22 °C, while the field frequency of 100 kHz was responsible for the smallest increase in temperature, which was 1.44 °C. The maximum temperature measured over the entire time span is 28.34 °C when the field frequency is 400 kHz; thus, the greatest amount of heat was generated at 400 kHz during the 10-min exposure period. ∆T/∆t is the sole variable in the above equation, hence SAR is proportional to the rate of temperature change or the slope of the lines in Fig. 8a. Since there are two time periods, it is possible to compute two SAR values for each example. Figure 8b displays SAR values as a function of field frequency for two time periods. Based on Fig. 8a, we can expect that the first time period at 300 kHz will have the highest SAR value because its graph is steeper. The SAR values in the first time interval at 100 kHz, 200 kHz, 300 kHz and 400 kHz are 2.7 (W/g), 41.2 (W/g), 33.2 (W/g), and 33.2(W/g), increasing as the field frequency rises. In the second 5 min, it’s interesting to see how the temperature goes in a different direction. In the second time interval, the SAR at a field frequency of 100 kHz is 24.2 W/g, which is more than ten times the value in the first time interval. In the SAR, after 100 kHz, the second time interval’s lowest SAR value of 12.6 (w/g) at 300 kHz is seen. For the first, second, and whole time intervals, the mean values of SAR are determined as 27.57 (W/g), 22.7 (W/g), and 23.8 (W/g), respectively.

In order to ensure the reliability of the data, the experimental tests were repeated for each field frequency and the temperature was recorded initially and every 5 min in a 10-min period. the mean values of the measured temperatures were used to draw the heating profiles and calculate the SAR value. The maximum standard deviation reported was 0.53 ℃ for a set of recorded temperatures depicted in Table 1.

**Table 1:** A set of recorded temperatures in different frequencies and a 10-min period.

Entry	f = 100KHZ			f = 200KHZ			f = 300KHZ			f = 400KHZ		
	T_0_	T_1_	T_2_	T_0_	T_1_	T_2_	T_0_	T_1_	T_2_	T_0_	T_1_	T_2_
1	25.25	25.30	26.87	24.45	26.46	27.89	26.27	27.13	27.76	25.53	27.30	28.36
2	24.56	24.95	25.96	23.94	26.12	27.57	25.67	26.67	27.41	24.91	26.46	27.74
3	25.64	26.07	27.12	25.11	27.10	28.78	26.96	27.86	28.53	26.06	27.72	28.92
Mean	25.15	25.44	26.65	24.5	26.56	28.08	26.3	27.22	27.9	25.5	27.16	28.34
Standard Deviation	0.45	0.47	0.5	0.48	0.41	0.51	0.53	0.49	0.47	0.47	0.52	0.48

#### The evaluation of TG hydrogel/SF/Fe_3_O_4_ nanocomposite with former reports in hyperthermia process, MTT,and hemolysis assay

As previously mentioned, the efficiency and heating capabilities of the TG hydrogel/SF/Fe_3_O_4_ nanocomposite was determined using an alternating magnetic field (AFM). The lowest concentration of the nanocomposite (1 mg/mL) exhibited a maximum specific absorption rate (SAR) of 41.2 w/g, which is noteworthy. A variety of magnetic nanocomposites based on natural polymers such as chitosan, cellulose, alginate, and agar have been reported with varying optimal concentrations and distinct SAR values (Table 2). Upon comparison with the reports in Table 2, it was found that the TG hydrogel/SF/Fe_3_O_4_ nanocomposite exhibited notable and significant potential for use in hyperthermia processes due to its remarkable colloidal stability, high saturation magnetization (Ms), and substantial SAR values at the lowest possible concentration (1 mg/mL). Therefore, these types of nanocomposites can be considered stable and recyclable magnetic nanomaterials for various applications. Furthermore, an extensive biological assessment was conducted on the nanocomposite fabricated using natural TG polymer, which included evaluations of cytotoxicity and blood compatibility (Table 3). The results demonstrate that the nanocomposite developed in this study exhibits superior blood cell compatibility compared to its counterparts (entry 3, 4, 7, and 8). This can be attributed to the incorporation of low-toxicity constituents from natural TG polymer, as well as SF protein strands that envelop Fe_3_O_4_ magnetic nanoparticles in a thick shell. Furthermore, the level of cytotoxicity observed in the prepared nanocomposite is significantly lower than that of other TG polymer-based nanocomposites (entry 1, 2, 3, 5, 6, and 8).

**Table 2:** The comparison of TG hydrogel/SF/Fe_3_O_4_ nanocomposite with other reported resarches in hyperthermia process.

Entry	MNPs	Nanocomposite	The optimum concentration of nanocomposite (mg/mL)	SAR (w/g )	Magnetic saturation (emu/g)	References
1	Fe_3_O_4_	SA hydrogel/SF/HNTs/Fe_3_O_4_	5	22.3	15.96	^53^
2	Fe_3_O_4_	CN-CMC hydrogel/SF/Fe_3_O_4_	1	7	38.0	^58^
3	Fe_3_O_4_	Magnetic chitosan-*g*-poly(*N*-vinylcaprolactam)	2	~ 204	~ 37	^59^
4	Fe_3_O_4_	MNHG-1-Fe	20	25	11	^60^
5	Fe_3_O_4_	DOX-Fe_3_O_4_@agar	–	~ 18.9	41.9	^61^
6	Fe_3_O_4_	TG hydrogel/SF/Fe_3_O_4_	1	41.2	48.76	This work

**Table 3:** The comparison and evaluation of TG hydrogel/SF/Fe_3_O_4_ nanocomposite with other reported.

Entry	Natural polymer	Nanocomposite	MTT(%)	Hemolysis (%)	Time (h)	Ref
1	TG	EC-loaded nanogel	81	–	72	^62^
2	TG	TG-*g*-PNIPAAm/Fe_3_O_4_	93	–	24	^63^
3	TG	FSRMH	93.6	~ 2.4	24	^64^
4	TG	TG-co-SA-cl-PVA	–	~ 0.83	24	^65^
5	TG	GT-GO5	~ 90	–	72	^36^
6	TG	Hydrogels 1 and 2	~ 93	–	24	^63^
7	TG	TG-*g*-PANI)S3)	121.6	< 2	72	^66^
8	TG	TG hydrogel/SF/Fe_3_O_4_	95.53	No hemolytic effect	72	This work

## Conclusions

This study presents the fabrication of a novel magnetic nanobiocomposite for hyperthermia cancer therapy, using TG hydrogel and SF protein as its base materials. The TG structure contains hydroxyl and carboxyl groups that can create a three-dimensional hydrogel network in the presence of divalent Ca^2+^ ions. The ionic cross-linking agent was preferred over more toxic organic monomers due to its lower toxicity. The resulting TG hydrogel/SF hybrid was magnetized with Fe_3_O_4_ MNPs for hyperthermia application. The natural components of the nanocomposite, such as TG and silk fibroin, are suitable for coating Fe_3_O_4_ MNPs due to their hydrophilic functional groups and swelling properties, which prevent their premature sedimentation. Moreover, the inclusion of SF protein in the nanobiocomposite structure increases its biocompatibility, biodegradability, strength, and cell adhesion, making it suitable for biological applications like tissue engineering, wound healing, and drug delivery.

To ensure that the fabricated nanocomposite is safe for biological applications, hemolysis, and MTT tests were performed to investigate its cytotoxicity. The results demonstrate that the TG hydrogel/SF/Fe_3_O_4_ nanobiocomposite has no hemolytic effect and is non-toxic for healthy cells. Additionally, it exhibits anticancer activity (22.75%) against BT549 cells. The hyperthermia application of this magnetic nanobiocomposite was evaluated, and the maximum SAR value was measured (41.2 W/g) at 200 kHz. Based on all the results obtained, this study introduces the nanobiocomposite as a multi-functional system for hyperthermia cancer therapy. Furthermore, it can be a promising candidate for drug delivery, tissue engineering, and wound healing in future studies.

## Data Availability

All data generated or analysed during this study are included in this published article.
